# Supplementary figures and images for: Opposite asymmetries of face and trunk and of kissing and hugging, as predicted by the axial twist hypothesis
Source: PeerJ. 2019 Jun 7;7:e7096. doi: 10.7717/peerj.7096 (PMC6557252; doi:10.7717/peerj.7096)

**3D analysis**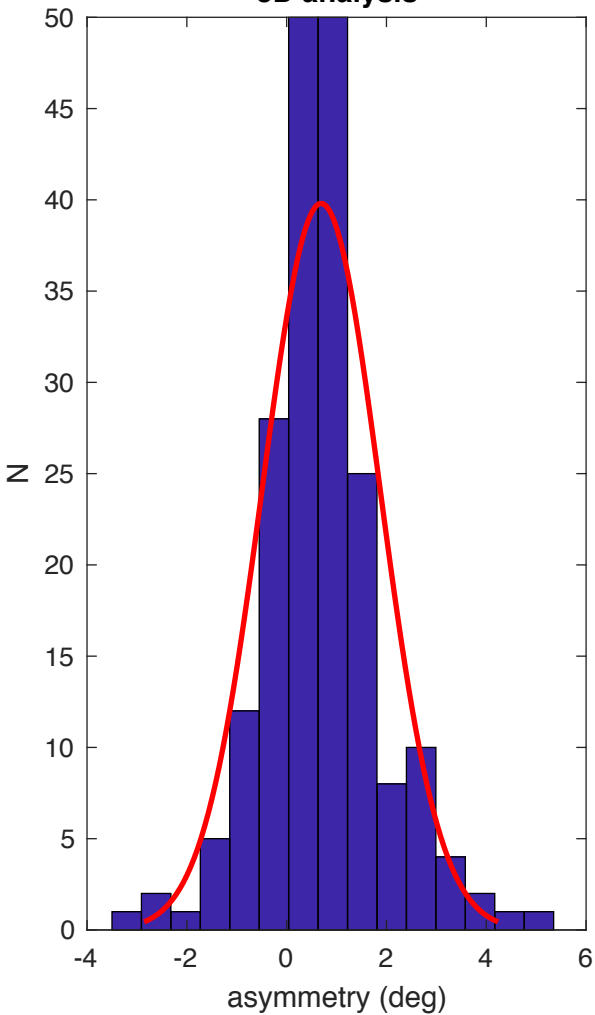**Picture analysis**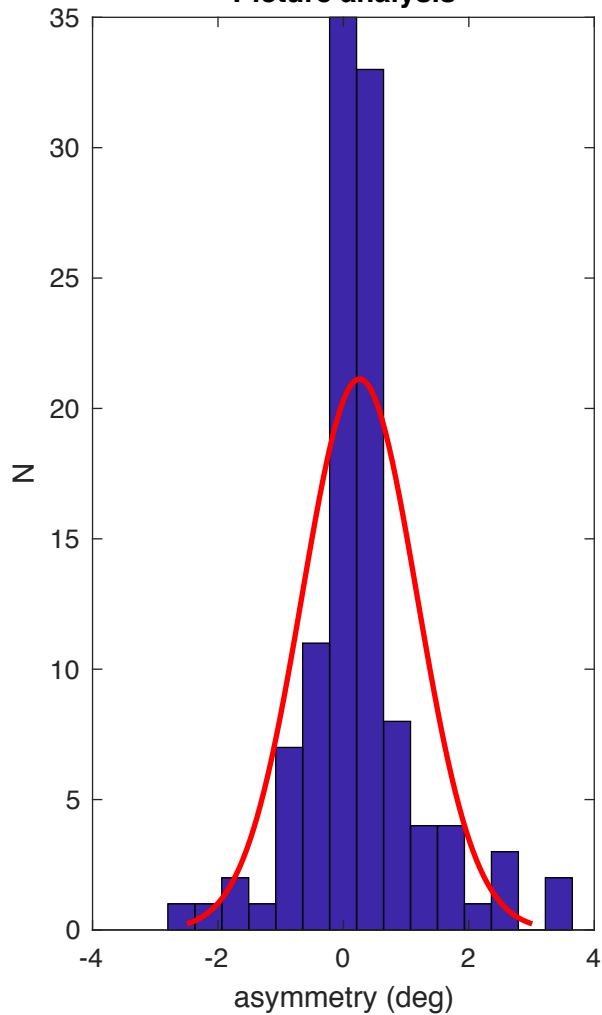

Supplement: Figure S1 — Left: for the 3D database (Troje & Bülthoff, 1996), and Right: for the 2D analysis of frontal pictures of the face in a British population of mostly male subjects. Red curves: fitted normal distribution. [file peerj-07-7096-s001.pdf]

**correlation:  $r=0.93$**

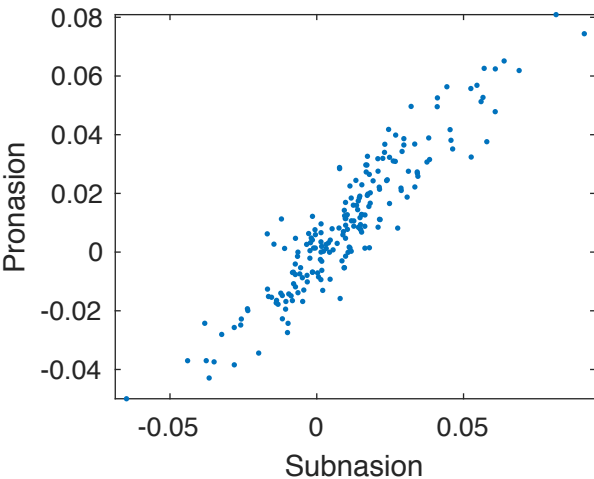

**correlation:  $r=0.83$**

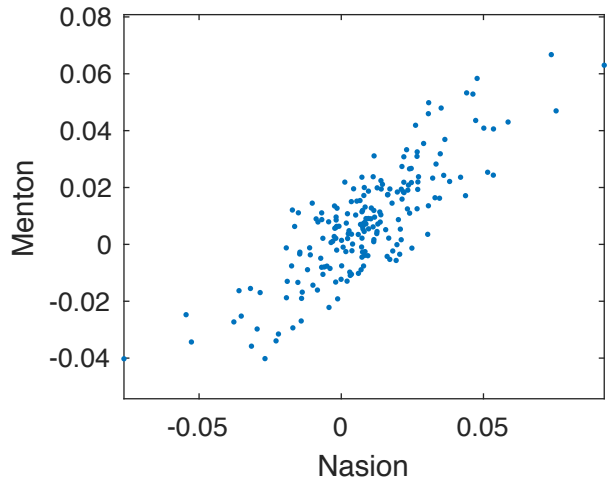

**correlation:  $r=0.86$**

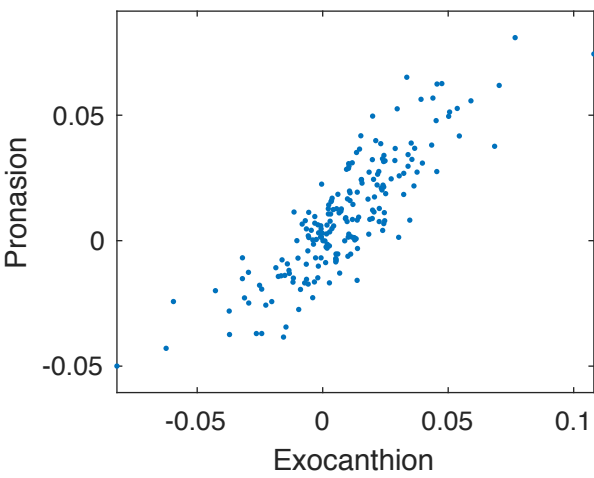

**correlation:  $r=0.96$**

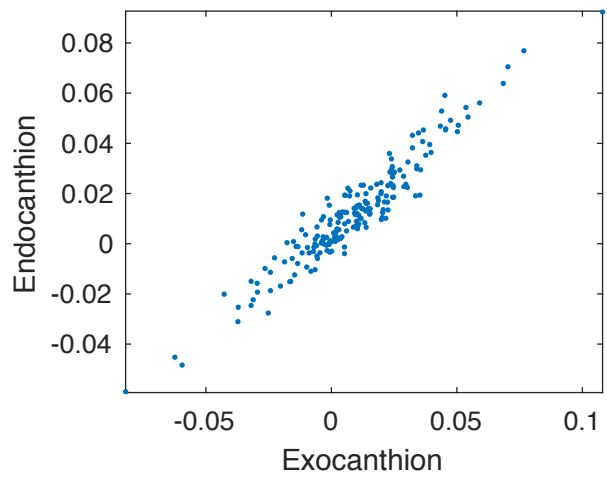

Supplement: Figure S2 — Each panel shows the correlation of two of the landmarks. The lowest (Nasion-Menton) and the highest (Exocanthion-Endocanthion) are the lowest and highest correlations respectively of all pairs of landmarks. [file peerj-07-7096-s002.pdf]
