# Supplementary material for: Opposite asymmetries of face and trunk and of kissing and hugging, as predicted by the axial twist hypothesis
Source: PeerJ. 2019 Jun 7;7:e7096. doi: 10.7717/peerj.7096 (PMC6557252; doi:10.7717/peerj.7096)
Supplement: Figure S3 — The aurofacial asymmetry was determined from the frontal photos (maximally one per subject). First, the photos were randomly flipped, then the eyes were horizontally aligned; the facial region was covered by an oval mask. Then the photos were selected that were truly frontal on the basis ear region. The masks were removed, and the eccentricity of the eyes was determined and expressed in degrees (see methods). These aurofacial eccentricities were written to the table at the end of the document, after the values for the flipped faces were multiplied by −1. [file peerj-07-7096-s003.pdf]

Analysis of the Aberdeen dataset. Fifty percent of the faces are flipped according to the pattern on the right side (green=not-flipped)

realigned: 11.7.2-16

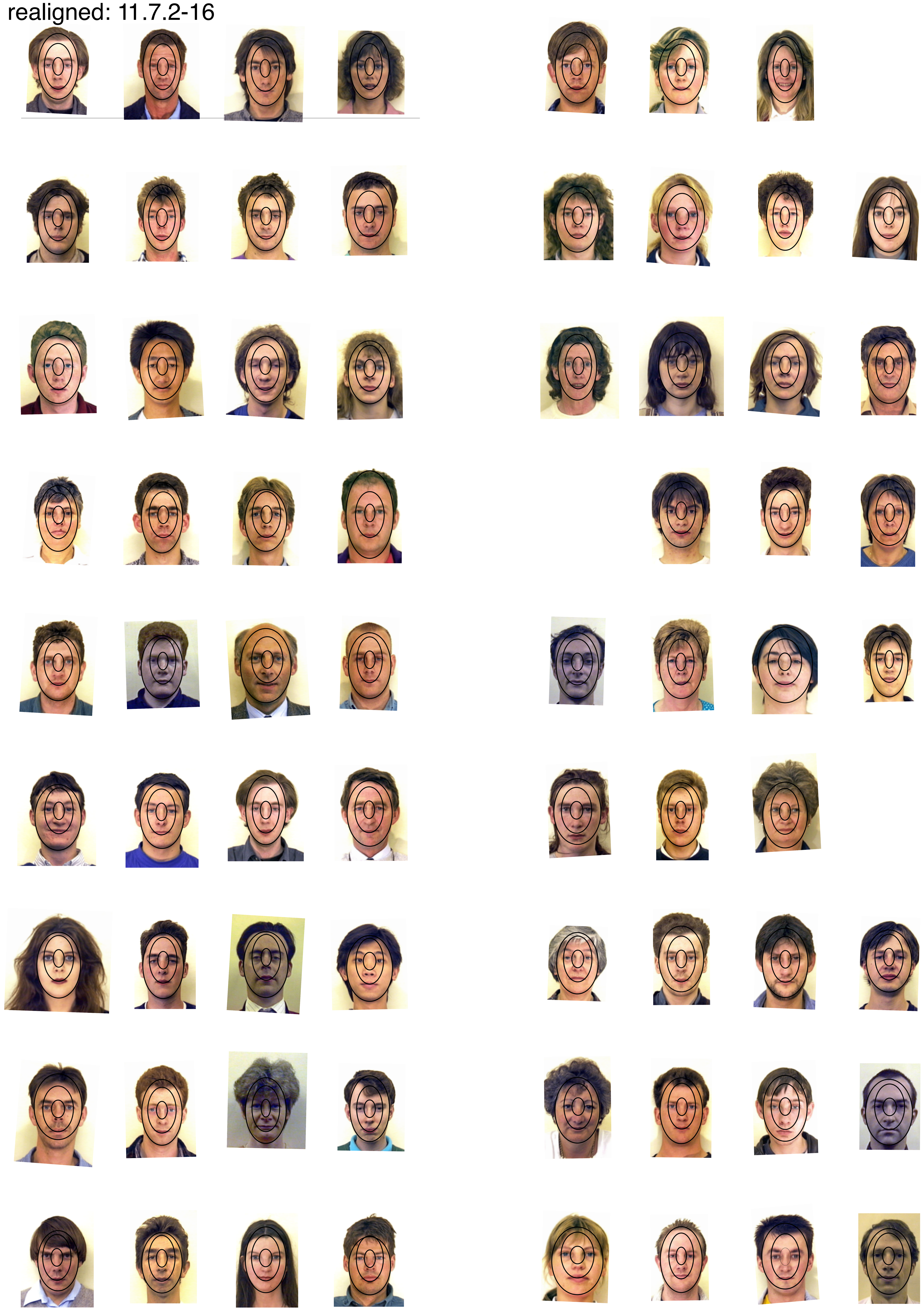

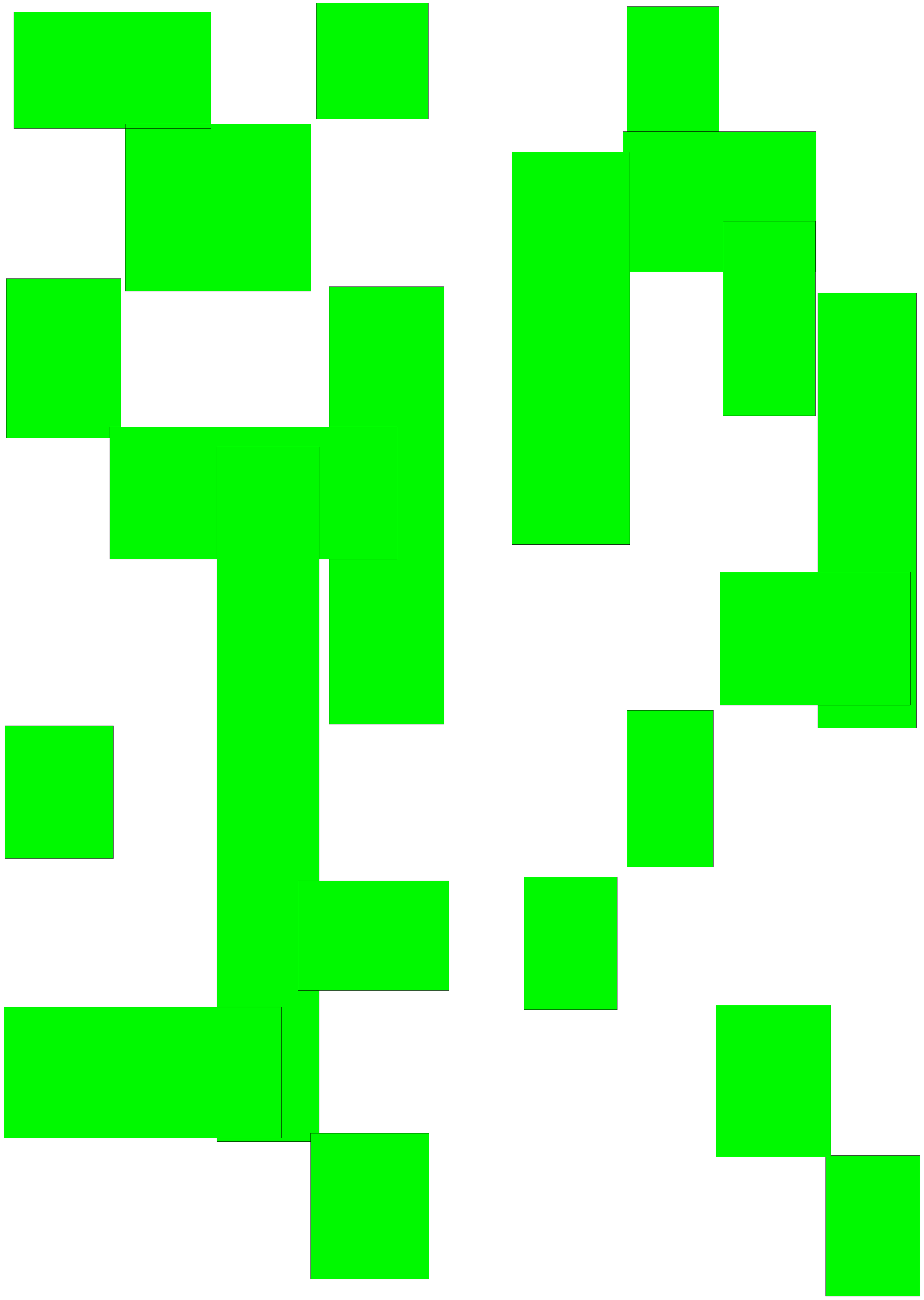

Analysis of the Nottingham dataset. Fifty percent of the faces are flipped according to the pattern on the right side (green=not-flipped)

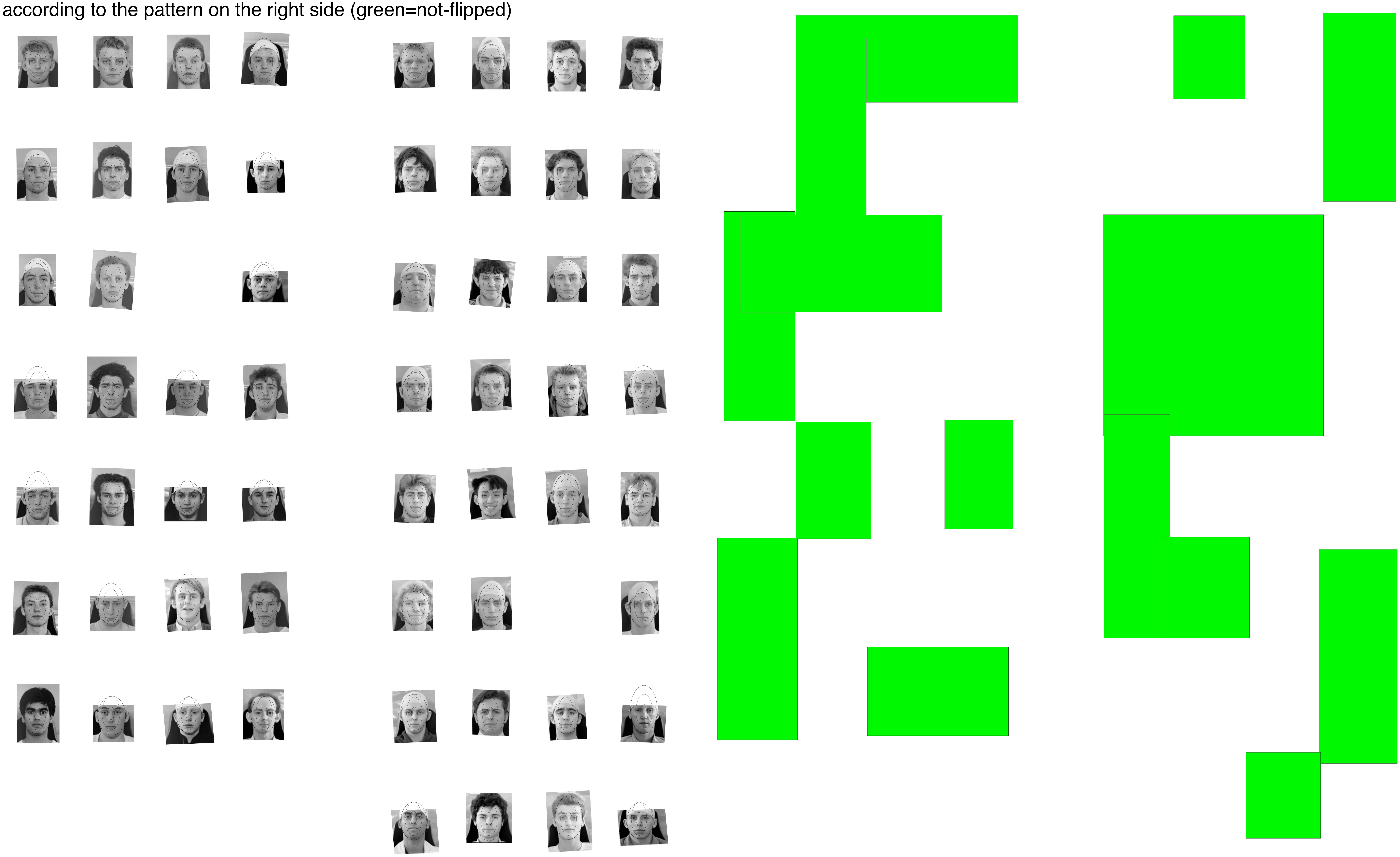

Aurofacial asymmetry of the facial photos

Dataset	Angle
**Aberdeen**	-2,52
**Aberdeen**	-2,09
**Aberdeen**	-1,85
**Aberdeen**	-1,52
**Aberdeen**	-1,25
**Aberdeen**	-1,06
**Aberdeen**	-1,01
**Nottingham**	-0,88
**Aberdeen**	-0,80
**Aberdeen**	-0,79
**Aberdeen**	-0,77
**Aberdeen**	-0,76
**Aberdeen**	-0,52
**Aberdeen**	-0,50
**Nottingham**	-0,45
**Nottingham**	-0,43
**Nottingham**	-0,41
**Nottingham**	-0,40
**Aberdeen**	-0,39
**Nottingham**	-0,29
**Nottingham**	-0,29
**Nottingham**	-0,28
**Nottingham**	-0,24
**Nottingham**	-0,12
**Nottingham**	-0,09
**Nottingham**	-0,06
**Nottingham**	-0,06
**Nottingham**	-0,06
**Aberdeen**	-0,01
**Aberdeen**	0,00
**Aberdeen**	0,00
**Aberdeen**	0,00
**Aberdeen**	0,00
**Aberdeen**	0,00

Dataset	Angle
**Aberdeen**	0,00
**Aberdeen**	0,00
**Aberdeen**	0,00
**Aberdeen**	0,00
**Aberdeen**	0,00
**Aberdeen**	0,00
**Nottingham**	0,00
**Nottingham**	0,00
**Nottingham**	0,00
**Nottingham**	0,00
**Nottingham**	0,00
**Aberdeen**	0,00
**Nottingham**	0,01
**Nottingham**	0,05
**Nottingham**	0,06
**Nottingham**	0,11
**Nottingham**	0,12
**Nottingham**	0,12
**Nottingham**	0,13
**Nottingham**	0,15
**Nottingham**	0,18
**Nottingham**	0,18
**Nottingham**	0,20
**Nottingham**	0,20
**Nottingham**	0,22
**Nottingham**	0,23
**Nottingham**	0,24
**Nottingham**	0,24
**Nottingham**	0,24
**Aberdeen**	0,25
**Aberdeen**	0,25
**Aberdeen**	0,25
**Nottingham**	0,28
**Nottingham**	0,28
**Nottingham**	0,28

Dataset	Angle
Nottingham	0,28
Nottingham	0,33
Nottingham	0,33
Nottingham	0,34
Nottingham	0,34
Nottingham	0,36
Nottingham	0,36
Aberdeen	0,38
Aberdeen	0,38
Aberdeen	0,38
Nottingham	0,44
Nottingham	0,45
Nottingham	0,46
Aberdeen	0,48
Aberdeen	0,50
Nottingham	0,51
Nottingham	0,51
Nottingham	0,52
Aberdeen	0,57
Nottingham	0,63
Aberdeen	0,63
Aberdeen	0,63
Nottingham	0,65
Nottingham	0,68
Nottingham	0,71
Aberdeen	0,80
Nottingham	0,81
Aberdeen	0,82
Nottingham	0,96
Aberdeen	1,06
Aberdeen	1,10
Aberdeen	1,21
Aberdeen	1,26
Aberdeen	1,26
Aberdeen	1,56

Dataset	Angle
**Aberdeen**	1,64
**Aberdeen**	1,77
**Aberdeen**	1,89
**Aberdeen**	2,26
**Aberdeen**	2,42
**Aberdeen**	2,52
**Aberdeen**	2,62
**Aberdeen**	3,37
**Aberdeen**	3,53
